# Sex-specific genetic analysis indicates low correlation between demographic and genetic connectivity in the Scandinavian brown bear (*Ursus arctos*)

**DOI:** 10.1371/journal.pone.0180701

**Published:** 2017-07-03

**Authors:** Julia Schregel, Alexander Kopatz, Hans Geir Eiken, Jon E. Swenson, Snorre B. Hagen

**Affiliations:** 1 Norwegian Institute of Bioeconomy Research, NIBIO - Svanhovd, Svanvik, Norway; 2 Norwegian University of Life Sciences, Faculty of Environmental Sciences and Natural Resource Management, Ǻs, Norway; 3 Norwegian Institute for Nature Research, Trondheim, Norway; National Cheng Kung University, TAIWAN

## Abstract

The degree of gene flow within and among populations, i.e. genetic population connectivity, may closely track demographic population connectivity. Alternatively, the rate of gene flow may change relative to the rate of dispersal. In this study, we explored the relationship between genetic and demographic population connectivity using the Scandinavian brown bear as model species, due to its pronounced male dispersal and female philopatry. Thus, we expected that females would shape genetic structure locally, whereas males would act as genetic mediators among regions. To test this, we used eight validated microsatellite markers on 1531 individuals sampled noninvasively during country-wide genetic population monitoring in Sweden and Norway from 2006 to 2013. First, we determined sex-specific genetic structure and substructure across the study area. Second, we compared genetic differentiation, migration/gene flow patterns, and spatial autocorrelation results between the sexes both within and among genetic clusters and geographic regions. Our results indicated that demographic connectivity was not a reliable indicator of genetic connectivity. Among regions, we found no consistent difference in long-term gene flow and estimated current migration rates between males and females. Within regions/genetic clusters, only females consistently displayed significant positive spatial autocorrelation, indicating male-biased small-scale dispersal. In one cluster, however, males showed a dispersal pattern similar to females. The Scandinavian brown bear population has experienced substantial recovery over the last decades; however, our results did not show any changes in its large-scale population structure compared to previous studies, suggesting that an increase in population size and dispersal of individuals does not necessary lead to increased genetic connectivity. Thus, we conclude that both genetic and demographic connectivity should be estimated, so as not to make false assumptions about the reality of wildlife populations.

## Introduction

The viability of a species is heavily influenced by the genetic connectivity within and among populations [1]. Gene flow is one of the main determinants of population genetic structure and retention of genetic diversity. Therefore, knowledge about gene flow is important for wildlife conservation and management, e.g., to outline rescue plans for threatened species, improve inter-population connectivity, and predict impacts of climatic change, biological invasions, and anthropogenic disturbances [2–6]. Demographic connectivity, i.e., dispersal, does not necessarily translate into gene flow, i.e., genetic connectivity [7, 8], which is why the application of genetic methods to assess effective dispersal, i.e., dispersal that leads to gene flow, is increasing rapidly [9]. However, few studies compare genetic and demographic connectivity and little is known about the interplay between these processes. Whether gene flow correlates with dispersal or not is a potentially important question, not only in wildlife conservation and management, but also in evolutionary biology and ecology.

In this study, we have explored the relationship between genetic and demographic connectivity using the brown bear (*Ursus arctos*) as a model species. Because of a successful comeback after near extinction [10, 11], the brown bear in Northern Europe has been used in several large-scale genetic studies of population connectivity, recovery, and range expansion [12–14]. Brown bears exhibit both long- and short-distance dispersal [15] and radio-telemetry studies have shown that the species exhibits male-biased dispersal, with subadult male dispersers emigrating at higher rates and across larger distances than female dispersers [15–17]. A genetic analysis has shown that females tend to form matrilineal assemblages, with a positive correlation between relatedness and home range overlap [18]. It follows, thus, that the degree of gene flow may be dependent on sex in the brown bear [19–21]. Accordingly, under the assumption that gene flow closely tracks dispersal, philopatric females should shape genetic population structure locally, whereas dispersing males should act as genetic mediators among regions. To test this empirically, we contrasted sex-specific population genetic parameter estimates both within and among genetic clusters with demographic data.

To this aim, we performed a sex-specific analysis based on STR (Short Tandem Repeats, i.e., microsatellites), using the genotypes of 1531 brown bears obtained in the course of the national and regional monitoring programs in Sweden and Norway. The spatial extent of sampling covered the entire distribution of the Scandinavian brown bear population with a high sampling density. We tested whether male and female population genetic structure differed, both regionally and locally, and whether dispersal and gene flow estimates reflected the documented pattern of male-biased dispersal and female philopatry both within and among genetic clusters and geographic regions. This approach allowed us to evaluate whether dispersal was a useful indicator of gene flow.

## Material and methods

### Sampling and genetic analysis

Brown bears in Norway and Sweden are monitored by noninvasive genetic sampling of mostly feces, but also hair. For this study, we utilized the 1461 georeferenced and unambiguous genotypes (707 females, 754 males) obtained in the course of this monitoring between 2006 and 2013. In addition, we included 70 genotypes (35 males and 35 females) from legally shot bears from Jämtland in Sweden that originated from 2005–2013, with the exception of three females from 1995, 1998 and 1999, and two males from 2003 and 2004. The entire study area reached from ~60°-70°N and from ~8°-30°E, encompassing ~457000km^2^ and the longest distance being ~1325km. The tissue samples were provided by the National Veterinary Institute of Sweden. No ethics permissions were required, as sample collection did not involve live animals and was performed by the respective national monitoring authorities of Sweden and Norway.

Eight validated microsatellite markers (MU09, MU10, MU23, MU59, MU05, MU51, MU50 [22] and G10L [23–25]) were used for genotyping, which followed a strict analysis protocol accredited according to the EN ISO/IEC 17025 standard and which has been described in detail previously [26].

### Statistical analysis of genetic population structure

We analyzed genetic population structure using STRUCTURE v.2.3.4 [27, 28] in a hierarchical manner to identify both, genetic clusters and substructure within clusters. Assuming population admixture and correlated allele frequencies, we set the maximum number of populations to K = 10 with ten independent runs for each K. The burn-in period was 100,000 Markov-Chain-Monte-Carlo (MCMC) iterations with a subsequent sampling of 1,000,000 MCMC iterations. We processed the results using Structure Harvester [29], which implements the *ad hoc* approach of Evanno et al. [30], and assigned the individuals to one of the inferred clusters, using a membership value of q≥0.7 as a threshold value [13, 31]. We reanalyzed each inferred cluster separately to test for additional substructure. In this analysis, we set the maximum number of inferred populations to K = 5; all other parameters were as described above.

### Estimation of scale of isolation by distance and assessment of fine-scale genetic structure

We analyzed genetic differentiation between pairs of individuals in a hierarchical manner by estimating the relationship between genetic and geographic distance on the small, i.e., within genetic clusters, and the large scale, i.e., across the entire sampling area. Across the entire sampling area, we used the kinship coefficient by Loiselle et al. [32], implemented in the program SPAGeDi v.1.4c [33]. This coefficient is supposed to suffer less bias in the presence of low allele frequencies [32] and has low sampling variance [34]. We did this using a distance class size of 40 km.

Within clusters, we performed a spatial autocorrelation analysis using GenAlEx 6.501 [35, 36], which also offers a heterogeneity test for the detection of sex-biased dispersal and has been shown to work well [37, 38]. A within-cluster analysis may potentially underestimate the strength of the genetic-geographic distance relationship by excluding admixed individuals and first-generation migrants. To test whether this was a problem in our approach, we reran the analysis based on pooling the individuals according to sampling location into five regions (S6 Fig). Similarly, pooling data from spatially distant and genetically differentiated populations may potentially inflate the genetic correlation coefficient, *r*, of neighboring samples, because genetic distances between individuals across the genetically differentiated sampling area are comparably much larger [38]. To tackle this issue within the clusters where we found substantial substructure, we reran the analysis of spatial autocorrelation using the multiple-populations-approach [39], treating the detected subclusters and admixed individuals as population units.

### Genetic diversity within and differentiation among clusters

We calculated the number of alleles and observed and expected heterozygosity using GenAlEx 6.501 [35, 36] and inbreeding coefficient using Genetix 4.05.2 [40]. For the estimation of population differentiation, it is recommended to estimate several different estimators and execute caution in their interpretation in order to avoid erroneous conclusions [41–44]. Following this recommendation, we used GenAlEx 6.501 [35, 36] to estimate F_ST_, G_ST_ [45–47], G’_ST_ [48], and D [49]. The program calculates G_ST_ using the corrections proposed by Nei & Chesser [47], calculates G’_ST_ according to Hedrick [48] and follows the formulae given in eq.2 in Meirmans & Hedrick [42] to calculate D_est_ [35, 36]. To test for the dependency of G_ST_ on locus diversity, and thus gain insight on the influence of the high mutation rates of microsatellite markers on population differentiation, we used the software CoDiDi, developed by Wang [50].

### Effective migration among clusters

To estimate migration and gene flow among clusters, we used Genepop v4.0 [51], which implements the private allele method to estimate the number of effective migrants *N*_*m*_ [52] and corrects the estimate for number of samples by using a regression line, as described by Barton and Slatkin [53]. The private allele method is a global estimate, and thus may better reflect long-term rather than current gene flow, even though it is expected to react more quickly to an increase in gene flow rates than F_ST_-based estimates [54]. Therefore, we also applied the Bayesian software BAYESASS 3.0 [55], which is based on the population assignment method and is supposed to better reflect current migration and gene flow [56, 57]. We estimated the migration rates among geographic regions (see S6 Fig for regions) in ten independent runs using 21x10^6^ iterations with a burn-in period of 2x10^6^ iterations. We adjusted the delta values to 0.07 (allele frequency), 0.05 (inbreeding coefficient), and 0.15 (migration) and started each run with a random seed [56], as well as enabled the trace file option in order to test for convergence of the Bayesian estimations of migration rates afterwards [57]. Although being relatively free from assumptions, this method may have some limiting factors regarding the reliability of the results, such as low population structure and/or a high migration rate, leading to convergence problems [56]. Many empirical studies indeed show signs of convergence problems and it is recommended to perform multiple runs and estimate Bayesian deviance as a criterion to find the run with the best fit; we followed this recommendation [57].

### Dispersal as indicated by genetic monitoring records

In the monitoring database, each sample collected during the course of the monitoring action was recorded with the date and location of collection. Once the genetic analysis of the samples has been performed, the sample information was updated with an individual identifier. It is thus possible to retrace individual movements. As an indicator for dispersal among genetic clusters, we counted how many individuals in the monitoring database [58] were recorded in more than one county. We also counted how many individuals moved from one genetic cluster into the area of another and, as such, are potentially important genetic mediators among genetic clusters.

## Results

### Statistical analysis of genetic population structure

**Fig 1:**
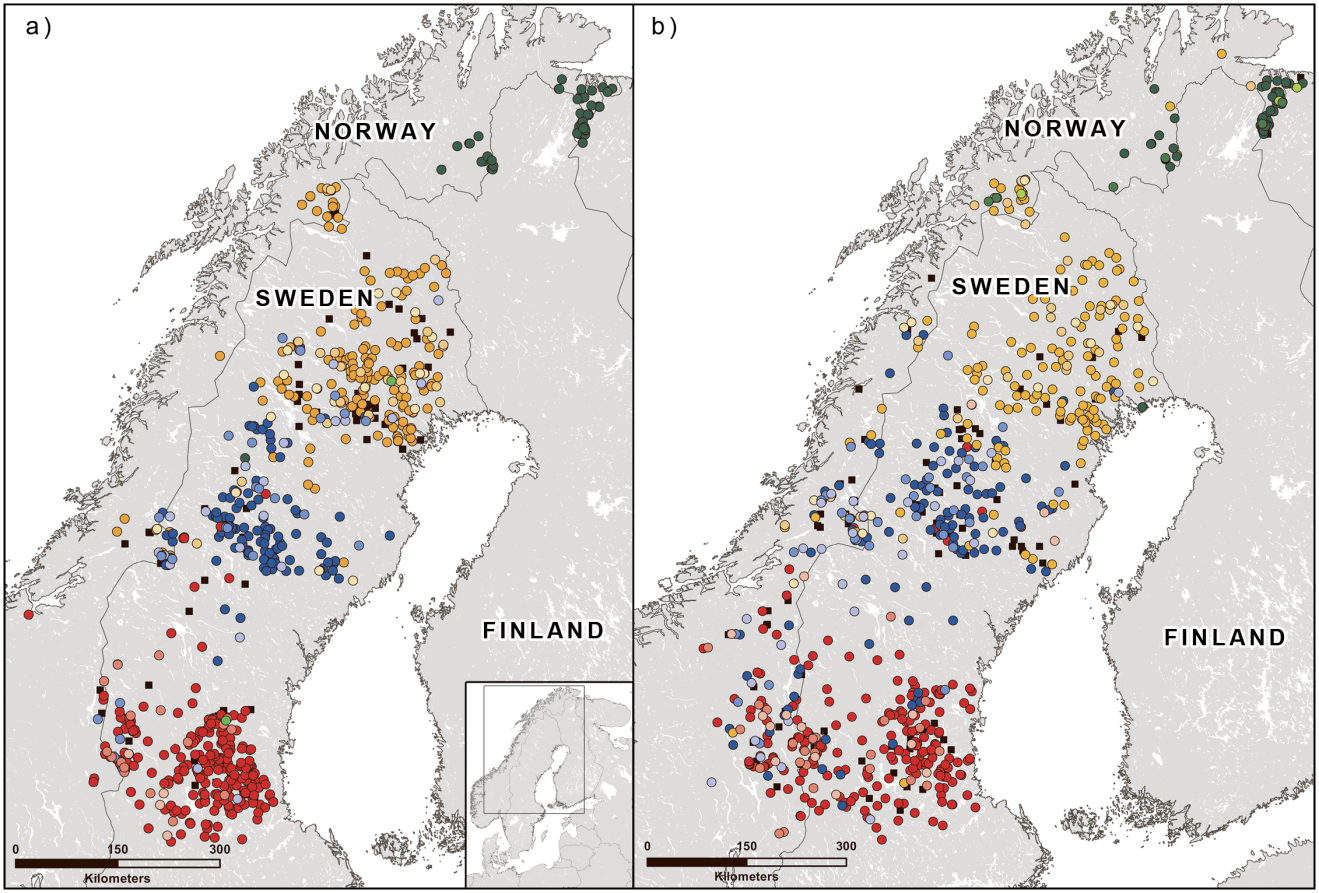
Location of genetic clusters for Norwegian and Swedish brown bears. a) only females, membership to a cluster is indicated by color, red = cluster 1, blue = cluster 2, yellow = cluster 3, green = cluster 4, darkest = membership value q ≥ 0.9; medium = q ≥ 0.8; lightest = q ≥ 0.7; black squares = q < 0.7; b) only males, colors correspond to a).

Based on the estimated likelihood values and Evanno’s ΔK, the initial STRUCTURE analysis suggested the existence of four genetic clusters across Norway and Sweden (Fig 1; S1–S2 Figs). Of these, three clusters previously documented only from Sweden using STRs were common for both countries. In addition, one cluster previously documented to be part of a larger Norwegian-Finnish-Russian cluster using autosomal and Y-STRs [13, 59, 60] was found only in northernmost Norway. Only a relatively small percentage of the individuals was not assigned to a genetic cluster: 10.9% of females, 13.3% of males, and 12.0% for females and males combined. We thus accepted these cluster assignments as having biological relevance and used them in the subsequent analyses. In the following, we refer to clusters 1 to 4 (from south to north; see Fig 1).

**Fig 2:**
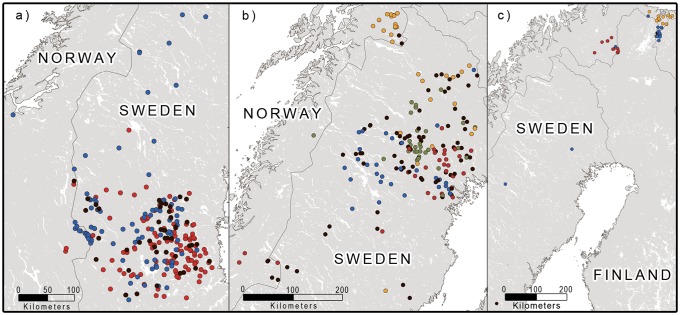
Location of female brown bears belonging to the different genetic subclusters in Scandinavia, determined by the reanalysis of each previously determined cluster. Individuals whose membership could not be determined (q < 0.7) are colored black. a) Subclusters within cluster 1, b) subclusters within cluster 3, c) subclusters within cluster 4. Note that the colors used to depict subcluster membership do not correspond to the color coding used in the other maps.

**Fig 3:**
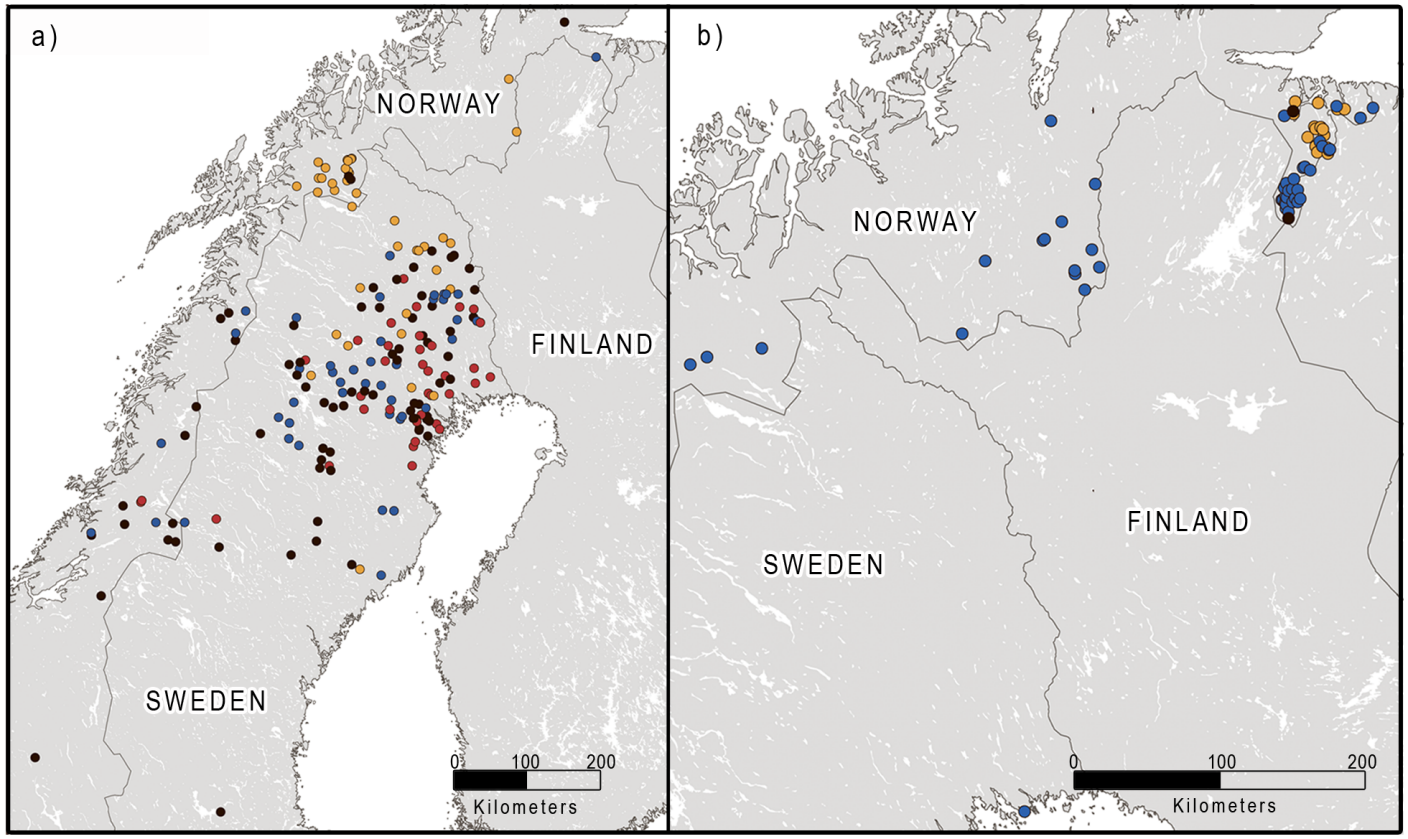
Location of male brown bears belonging to the different genetic subclusters in Scandinavia, determined by the reanalysis of each previously determined cluster. Individuals whose membership could not be determined (q < 0.7) are colored black. a) subclusters within cluster 3, b) subclusters within cluster 4. Note that the colors used to depict subcluster membership do not correspond to the color coding used in the other maps.

The second round of STRUCTURE analyses of each of the four clusters indicated additional genetic substructure within several of the clusters (Figs 2 and 3; S3–S5 Figs). In clusters 1 and 2, the results indicated a relatively weak genetic substructure, with only two overlapping subclusters for females in cluster 1. In clusters 3 and 4, the results indicated a relatively strong genetic substructure, with four and three relatively geographically coherent subclusters for both females and males, respectively. A relatively high percentage of the individuals was not assigned to a subcluster, compared to a cluster (17.6% for females in cluster 1, 41.1% for females and 38.8% for males in cluster 3, 4.2% for females and 3.1% for males in cluster 4), consistent with a weaker genetic structure within than among the clusters.

### Estimation of scale of isolation by distance and assessment of fine-scale genetic structure

**Fig 4:**
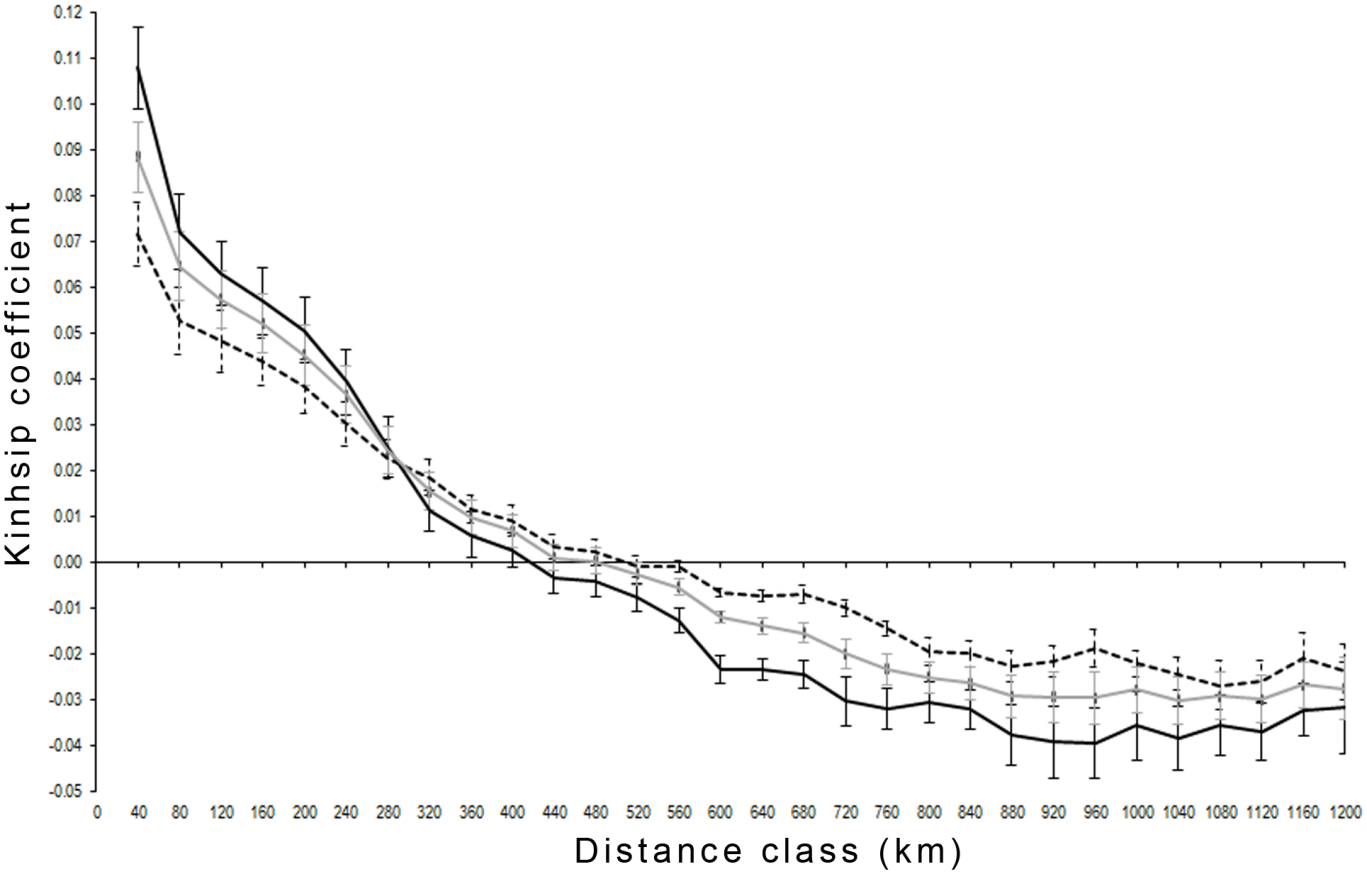
Loiselle kinship coefficient for brown bears in Scandinavia. One distance class is 40 km, solid black line = females; broken black lines = males; solid gray lines = females and males combined; 95% confidence intervals are displayed accordingly.

On a large geographical scale across the entire sampling area, the results showed a typical isolation-by-distance (IBD) pattern with a significantly decreasing kinship coefficient with increasing geographic distance for both sexes (Fig 4). Females displayed a slightly steeper slope than males and higher kinship coefficients in each distance class up to 280 km, after which their coefficients dropped below the male kinship coefficients.

**Fig 5:**
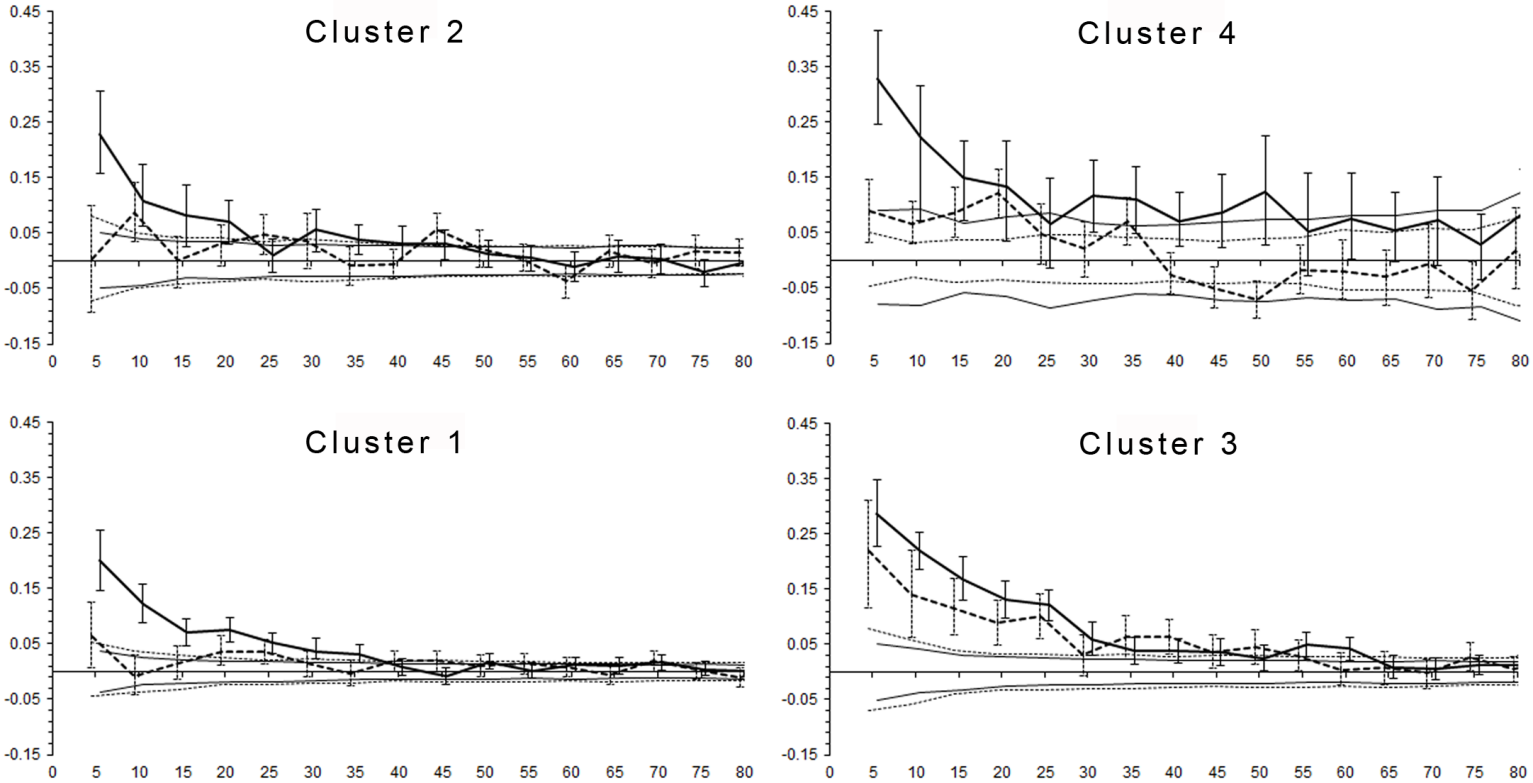
Spatial autocorrelation of female versus male brown bears compared within each cluster in Scandinavia. Distance class of 5 km (x axis); the genetic correlation coefficient (*r*, y-axis) is given as a solid line for females and a dashed line for males. The 95% confidence intervals for the null hypothesis of random distribution of genotypes, as well as bootstrap errors, are displayed in the same manner.

On a small geographical scale, within the four genetic clusters, only females consistently displayed a decreasing genetic correlation coefficient (*r)* with increasing geographic distance (Fig 5). Significant *r* values for females were found up to 35 and 45 km in clusters 1 and 2, whereas in clusters 3 and 4, they were found up to 60 and 50 km, indicating a larger spatial extent of female philopatry in the two northernmost clusters compared to the two southernmost clusters. In comparison, males showed no significant *r* values in clusters 1 and 2, but a significant correlation between genetic and spatial distance in clusters 3 and 4 (Fig 5). Accordingly, the result of the heterogeneity test showed that females displayed significantly higher *r* values than males in clusters 1, 2, and 4, indicating sex-biased population structure locally. Within cluster 3, the heterogeneity test was nonsignificant, thus males and females did not show significantly different fine-scale population structure in this area. Running the analyses using a regional/geographic pooling of the data instead of the cluster-based approach had no substantial impact on the results, thus supporting the findings of our approach (S6 Fig).

**Fig 6:**
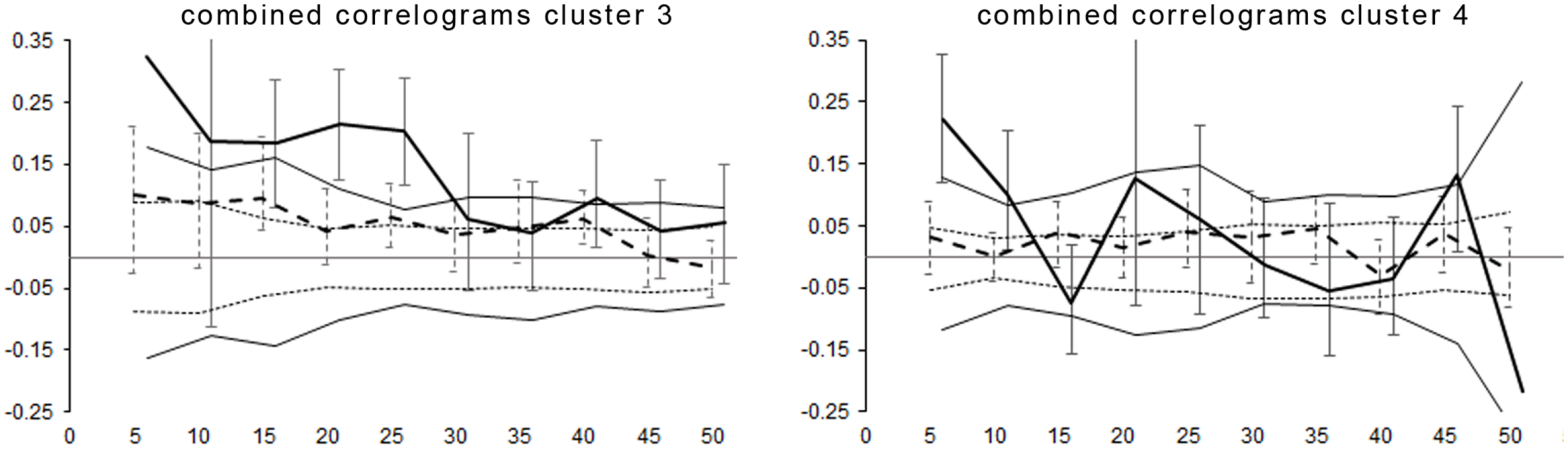
Combined spatial autocorrelation analysis for genetic clusters 3 and 4 for brown bears in Scandinavia. Distance class of 5 km (x axis); the analysis uses the multiple population approach, which sums the individual components for calculating *r*_*c*_ as a division of the total numerator and denominator across populations, rather than the simple arithmetic mean. The genetic correlation coefficient (*r*_*c*_, y axis) is given as a solid thick line for females and a dashed thick line for males. The 95% confidence intervals for the null hypothesis of random distribution of genotypes, as well as bootstrap errors, are displayed correspondingly in a thin line.

We reran the spatial autocorrelation analysis for clusters 3 and 4, using the multiple populations approach to control for the strong substructure found within these clusters. This yielded lower values of the genetic correlation coefficient compared to pooling the data of the entire cluster (Fig 6). However, males in cluster 3 still showed significantly positive spatial autocorrelation up to the 25-km distance class.

### Genetic diversity within and differentiation among clusters

**Fig 7:**
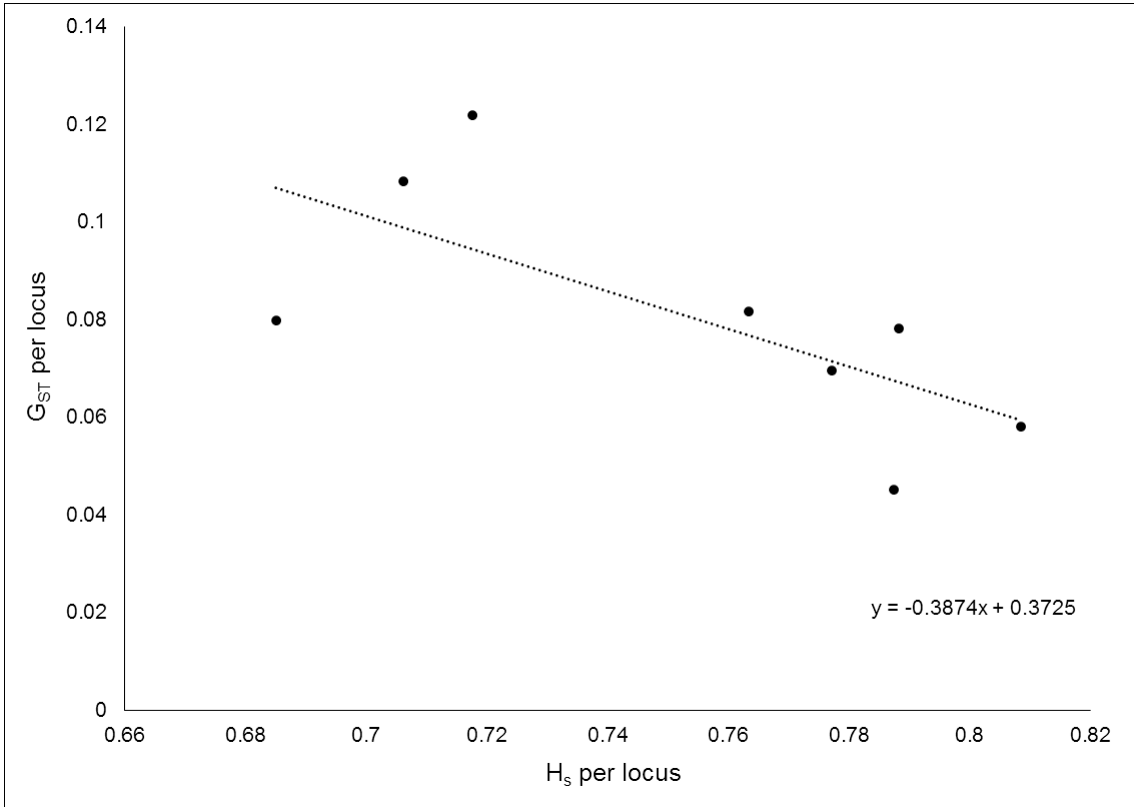
Result of the CoDiDi analysis showing the negative correlation between marker diversity and G_ST_ value in the Scandinavian brown bear population. The simple regression fitted through the data points is depicted as a dashed line, the slope of it is given in the graph. The p value of the correlation analysis was 0.053.

Genetic diversity, i.e. expected and observed heterozygosity, was high and >0.7 in all clusters and for both sexes (Table 1). Mean fixation index F_IS_ was between -0.056 and -0.005 and only one marker for females in cluster 3 showed a slightly higher positive and statistically significant value of 0.094 (Table 1). The relative magnitude of genetic differentiation among the four clusters was consistent among the different estimators F_ST,_ G_ST_, G’_ST_, and Joost's D_est_ (Table 2), as shown by the PCoA analysis (S7 Fig). For all four estimators, males displayed slightly lower values than females, with the exception of the pairwise G’_ST_ and D_est_ values between cluster 2 and 4, where males showed slightly higher values than females (Table 2). The results of the CoDiDi [50] analysis to evaluate the relationship between marker diversity and G_ST_ estimate showed a clear, if not statistically significant, negative correlation between the two (p value = 0.053) (Fig 7).

**Table 1:** Expected (H_e_) and observed heterozygosity (H_o_) and fixation index (F_IS_) per genetic cluster, estimated separately for female and male brown bears in Scandinavia.

	Locus	No. allele	H_e_	H_o_	F_IS_
**Cluster1**	MU09	6 / *6*	0.732 / *0*.*698*	0.739 / *0*.*700*	-0.010 / *-0*.*003*
	MU10	6 / *6*	0.804 / *0*.*804*	0.791 / *0*.*834*	0.016 / *-0*.*037*
	MU23	7 / *7*	0.716 / *0*.*665*	0.719 / *0*.*687*	-0.003 / *-0*.*032*
	MU59	7 / *9*	0.684 / *0*.*686*	0.719 / *0*.*684*	-0.051 / *0*.*004*
	MU05	6 / *6*	0.617 / *0*.*604*	0.625 / *0*.*656*	-0.013 / *-0*.*087*
	G10L	6 / *6*	0.720 / *0*.*731*	0.763 / *0*.*787*	-0.061 / *-0*.*076*
	MU51	6 / *5*	0.794 / *0*.*787*	0.782 / *0*.*787*	0.015 / *0*.*000*
	MU50	6 / *7*	0.758 / *0*.*748*	0.795 / *0*.*775*	-0.050 / *-0*.*035*
	**Mean**	**6.5 / *6*.*3***	**0.715 / *0*.*728***	**0.738 / *0*.*742***	**-0.033 / *-0*.*020***
**Cluster2**	MU09	9 / *10*	0.803 / *0*.*825*	0.775 / *0*.*838*	0.035 / *-0*.*016*
	MU10	7 / *7*	0.685 / *0*.*660*	0.689 / *0*.*667*	-0.006 / *-0*.*010*
	MU23	7 / *8*	0.817 / *0*.*825*	0.870 / *0*.*820*	-0.065 / *0*.*006*
	MU59	9 / *10*	0.781 / *0*.*790*	0.764 / *0*.*837*	0.022 / *-0*.*059*
	MU05	6 / *6*	0.633 / *0*.*631*	0.646 / *0*.*665*	-0.021 / *-0*.*053*
	G10L	7 / *8*	0.629 / *0*.*716*	0.609 / *0*.*678*	0.032 / *0*.*053*
	MU51	7 / *7*	0.721 / *0*.*749*	0.720 / *0*.*764*	0.000 / *-0*.*020*
	MU50	8 / *7*	0.815 / *0*.*819*	0.845 / *0*.*828*	-0.036 / *-0*.*011*
	**Mean**	**7.9 / *7*.*5***	**0.752 / *0*.*735***	**0.762 / *0*.*740***	**-0.014 / *-0*.*005***
**Cluster3**	MU09	8 / *9*	0.828 / *0*.*848*	0.872 / *0*.*933*	-0.053 / *-0*.*100*
	MU10	6 / *7*	0.793 / *0*.*779*	0.773 / *0*.*736*	0.024 / *0*.*056*
	MU23	8 / *7*	0.691 / *0*.*713*	0.665 / *0*.*705*	0.038 / *0*.*011*
	MU59	10 / *10*	0.831 / *0*.*835*	0.887 / *0*.*808*	-0.067 / *0*.*031*
	MU05	7 / *7*	0.754 / *0*.*755*	0.783 / *0*.*684*	-0.039 / *0*.094*
	G10L	8 / *8*	0.788 / *0*.*792*	0.729 / *0*.*860*	0.075 / *-0*.*086*
	MU51	7 / *7*	0.779 / *0*.*786*	0.823 / *0*.*833*	-0.056 / *-0*.*061*
	MU50	7 / *7*	0.746 / *0*.*728*	0.744 / *0*.*751*	0.002 / *-0*.*032*
	**Mean**	**7.8 / *7*.*6***	**0.779 / *0*.*776***	**0.789 / *0*.*784***	**-0.011 / *-0*.*009***
**Cluster4**	MU09	10 / *11*	0.829 / *0*.*867*	0.957 / *0*.*969*	-0.154 / *-0*.*118*
	MU10	6 / *8*	0.779 / *0*.*763*	0.681 / *0*.*810*	0.126 / *-0*.*061*
	MU23	7 / *9*	0.563 / *0*.*693*	0.574 / *0*.*688*	-0.021 / *0*.*008*
	MU59	11 / *11*	0.845 / *0*.*815*	0.830 / *0*.*767*	0.018 / *0*.*059*
	MU05	8 / *8*	0.829 / *0*.*802*	0.851 / *0*.*875*	-0.027 / *-0*.*091*
	G10L	5 / *6*	0.462 / *0*.*581*	0.511 / *0*.*656*	-0.106 / *-0*.*129*
	MU51	6 / *6*	0.786 / *0*.*787*	0.872 / *0*.*875*	-0.110 / *-0*.*111*
	MU50	7 / *8*	0.805 / *0*.*837*	0.787 / *0*.*844*	0.022 / *-0*.*008*
	**Mean**	**8.4 / *7*.*5***	**0.768 / *0*.*737***	**0.810 / *0*.*758***	**-0.056 / *-0*.*032***

Each cluster contains only individuals with a membership value q ≥ 0.7 as estimated by STRUCTURE. First value = females; second, italic value = males; bold = the mean across all loci for each cluster;

*) significant.

**Table 2:** Genetic differentiation among genetic clusters of Scandinavian brown bears assessed by F_ST_-, G_ST_-, G’_ST_- and Joost's D_est_-estimation.

		**Cluster 1**	**Cluster 2**	**Cluster 3**	**Cluster 4**
**Cluster 1**	**combined**		**0.276 /** 0.244	**0.463** / 0.425	**0.522** / 0.482
	**females**		**0.320 /** 0.285	**0.473 /** 0.435	**0.520 /** 0.479
	**males**		**0.244** / 0.215	**0.455** / 0.417	**0.528** / 0.489
**Cluster 2**	**combined**	**0.043 /** 0.042		**0.255** / 0.299	**0.459 /** 0.423
	**females**	**0.050 /** 0.049		**0.278** / 0.249	**0.448 /** 0.409
	**males**	**0.038** / 0.037		**0.240** / 0.215	**0.483** / 0.448
**Cluster 3**	**combined**	**0.066** / 0.066	**0.035 /** 0.035		**0.358 /** 0.327
	**females**	**0.067** / 0.066	**0.040 /** 0.038		**0.372 /** 0.339
	**males**	**0.066** / 0.067	**0.033** / 0.032		**0.362** / 0.331
**Cluster 4**	**combined**	**0.078** / 0.077	**0.065** / 0.064	**0.040 /** 0.046	
	**females**	**0.082** / 0.079	**0.070 /** 0.067	**0.054 /** 0.050	
	**males**	**0.079** / 0.077	**0.067 /** 0.065	**0.048** / 0.045	

Values below the diagonal: **F**_**ST**_ / G_ST_; values above diagonal: **G’**_**ST**_ / Joost's D_est_. All estimates have a p-value <0.05.

### Effective migration among clusters

Long-term gene flow, i.e., number of effective migrants per generation among the clusters using the private allele method, was surprisingly low (i.e. >1 in only two cases) and showed no consistent difference between males and females. (Table 3; S8 Fig). The BAYESASS estimates of migration rates used to assess current gene flow were generally also low and inconsistent between the sexes (Table 4). Overall, self-recruitment was high (>90%) in the South, North and Northeast, whereas it was lower in the central part; ~68% in South Central and ~85% in North Central (Table 4). The likelihoods of the run with the lowest deviance, which was chosen as the source for the estimates, is given in the supplementary material (S9 Fig). Estimates were consistent for both males and females across all ten runs (S10 and S11 Figs), except for females sampled in the northern part of Sweden, which showed slight variation between runs (S11 Fig).

**Table 3:** Number of migrants per generation among the four genetic clusters of brown bears in Scandinavia.

		**Cluster 1**	**Cluster 2**	**Cluster 3**
**Cluster 2**	**combined**	0.741		
	**females**	0.551		
	**males**	1.114		
**Cluster 3**	**combined**	0.081	0.814	
	**females**	0.107	1.309	
	**males**	0.168	0.729	
**Cluster 4**	**combined**	0.107	0.187	0.153
	**females**	0.144	0.253	0.205
	**males**	0.202	0.292	0.258

Estimated with the private allele method using Genepop v4.1.0

**Table 4:** Percentage of self-recruitment and directional migration/gene flow of brown bears in Scandinavia among regions.

From / To		**South**	**South Central**	**North Central**	**North**	**Northeast**
**South**	**females**	0.980 (0.006)	0.002 (0.002)	0.014 (0.006)	0.002 (0.002)	0.002 (0.002)
	**males**	0.925 (0.012)	0.011 (0.016)	0.058 (0.019)	0.006 (0.004)	0.001 (0.001)
**South Central**	**females**	0.134 (0.023)	0.686 (0.013)	0.162 (0.022)	0.014 (0.009)	0.004 (0.003)
	**males**	0.109 (0.020)	0.685 (0.024)	0.180 (0.046)	0.023 (0.012)	0.004 (0.004)
**North Central**	**females**	0.023 (0.013)	0.017 (0.009)	0.931 (0.021)	0.026 (0.011)	0.004 (0.004)
	**males**	0.055 (0.017)	0.035 (0.047)	0.830 (0.051)	0.079 (0.016)	0.002 (0.002)
**North**	**females**	0.016 (0.006)	0.159(0.013)	0.005 (0.003)	0.818 (0.013)	0.002 (0.002)
	**males**	0.004 (0.003)	0.010(0.008)	0.019 (0.012)	0.962 (0.013)	0.006 (0.004)
**Northeast**	**females**	0.007 (0.007)	0.008 (0.007)	0.009 (0.010)	0.008 (0.008)	0.968 (0.015)
	**males**	0.015 (0.009)	0.007 (0.007)	0.008 (0.007)	0.039 (0.015)	0.932 (0.019)

Estimates were performed with BAYESASS 3.0 [50]. Percentage of self-recruitment is given in the diagonal, shaded cells. Directional migration/gene flow among regions is presented above and below the diagonal. Standard deviations, as estimated by the software, are given in the brackets.

### Dispersal as indicated by genetic monitoring records

The monitoring database contained 48 females and 151 males that had been detected in more than one county, thus confirming more male than female dispersal movement. A total of 25 individuals (2 females, 23 males) moved from the area of one genetic clusters into the area of another, thus potentially mediating gene flow. However, the vast majority of these movements were between neighboring areas and only two males moved across a longer distance. One male moved ~ 430 km from Hedmark County in southeastern Norway, were it was recorded in 2007, to Västerbotten County in northern Sweden, were it was recorded in 2009, essentially moving out of the core area of cluster 1 and into the core area of cluster 2 (compare Fig 1b). This individual showed a *q* value of 0.554 for cluster 2. The other male moved ~ 400 km from Sør-Trøndelag County in central Norway in 2009 southwards to Telemark County in southern Norway, which is effectively free of bears, and was shot there in 2012. These records match the results of the radio-tracking studies performed previously in the Swedish bear population, where the average dispersal distances for 4-year-old males and females were 119 km and 28 km, respectively, whereas maximum observed dispersal distances were 467 km (males) and 90 km (females) [15].

## Discussion

Demographic and genetic connectivity are important factors for the survival of a population [61]. It is known that dispersal does not necessarily lead to gene flow, and here we have shown that generalizing about gene flow from information about dispersal behavior based on radio-tracking studies may be problematic. Our results show that dispersal patterns and the degree of differentiation between male and female dispersal behavior in brown bears varies with region, especially in northern Sweden, where, contrary to our expectation, males did not display random distribution across the landscape and rather showed a pattern of philopatry similar to females. At the same time, our monitoring records indicated that large-scale movement among genetic clusters may be fairly low and thus gene flow across the study area may be limited.

### Genetic population structure

Previous studies in Scandinavia have shown 3 to 4 distinct genetic clusters, which were regarded as being caused by the historic bottleneck event and/or genetic drift [62, 63], indicating a time-lag effect [64]. At the highest hierarchical level, our STRUCTURE analysis corresponded well with these studies, but at a finer resolution it also showed, for the first time, latitudinal variation both in the scale and sex-bias of genetic structuring increasing towards the North. A time-lag effect may explain some of the observed population genetic structure. However, most knowledge about the temporal scale of shifts in population genetic structure in expanding populations, due to founder effects and/or bottleneck events, is based on simulation studies (e.g. [64]). Only a limited number of empirical studies have been published and they showed both long-lasting effects on population structure (e.g. [65, 66]) and relatively quick changes in population differentiation (e.g. [67, 68]) in founder effect settings. In contrast to this, admixture and a decrease in population differentiation progressed on a short temporal scale, i.e., 1.5 generations, in the Finnish brown bear population with a similar population recovery setting as in Sweden and Norway [14]. In Sweden, male brown bears regularly disperse across long distances (average male natal dispersal distance = 118.9 km; [15]), which should aid gene flow among regions and a rapid decrease of population genetic structure.

Our data further suggested that there was an area effect regarding the degree of genetic population structure, with the southern regions being less structured than the northern ones. This kind of directional trend is often observed in expanding populations, with an increasing population differentiation towards the expansion front [69–71]. However, this explanation can be ruled out for the Scandinavian population, as population expansion did not occur unidirectionally, but from several different refugia [10, 62], with the northern area representing the part of the population that was least affected and recovered much faster than the more southern areas [10]. Thus, our results suggest that ecological and/or behavioral mechanisms restrict gene flow among clusters.

### Fine-scale genetic structure and sex-biased dispersal

In accordance with Støen et al. [15], our analysis of spatial autocorrelation indicated female philopatry brown bears. For most of the areas, the results also indicated a random distribution of male brown bears, in agreement with male-biased dispersal. However, in contrast to this, we found significant positive spatial autocorrelation for males in cluster 3, even after applying the multipopulation approach to account for high levels of within-cluster structuring [35, 39]. Upwards bias of the genetic correlation value *r* could occur under strong isolation-by-distance, where the most distant subgroups exhibit the largest degree of genetic differentiation. However, the detected subgroups showed a relatively large degree of spatial overlap and genetic difference could not be explained by spatial distance. Instead, our results suggest the presence of mechanisms that support the formation of family clusters for both sexes, such as kin recognition and territorial behavior, which may lead to a lower acceptance of nonkin immigrants [66]. Such behavior is generally associated with philopatric females [15, 18] and we are not aware of studies suggestive of kin cooperation among male brown bears. Indications of a stronger substructure in the northern parts of the population have been found in previous studies and were explained by the influence of matrilineal formations [62, 63]. Our results suggest that this is not the entire picture and that also patrilineal genetic patterns may be involved in the increased substructure in the North.

Another possible explanation is the impact of illegal killing, which occurs more frequently and is more widely accepted in the North than in the South and has a strong effect on large carnivore populations [72–76]. Male bears leave the dens earlier than females, often at a time when there is still snow on the ground, enabling poachers to use snowmobiles and thus kill more effectively [76]. One can speculate that this may either hinder effective dispersal out of the natal home range by the death of young, dispersing males or by the bears adapting their dispersal behavior to favor not dispersing far from their natal home range.

### Population differentiation among clusters

There is still a lot of uncertainty and confusion about which measure of differentiation is more correct in which situation (e.g., [77–82]). G_ST_ is supposed to describe the influence of demographic events on the population structure better than D_est_, which is supposed to better reflect historic population/colonization events [81, 83]. In practice, though, G_ST_ seems to underestimate differentiation relative to D_est_ often, but reaches equilibrium much more quickly [42]. Generally, F_ST_ is still seen by many as the base standard, which always should be included in a study on genetic differentiation [42, 80, 82]. In our study, all pairwise differentiation estimates were relatively congruent for the different estimators, which may point to a relatively stable population structure and gene flow pattern over time, so that the proposed differences in what the estimates measure had little impact on our population differentiation estimation in the Scandinavian population. However, the observed negative correlation of marker diversity with the G_ST_-value points to a higher mutation than migration rate, which leads to an overall increase of diversity, causing G_ST_ and similar measures to underestimate population differentiation [84]. In addition, even though the result was not statistically significant, the slope of the correlation was clearly negative, so that we should expect that a different set of markers with different levels of diversity may give different results [50]. This indicates that one should be careful to draw definite conclusions about the degree of differentiation and its underlying causes based only on the F_ST_-based estimators.

### Dispersal behavior versus gene flow

The monitoring database confirmed more male than female dispersal movement. These records match the results of the radio-tracking studies performed previously in the Swedish bear population, where the maximum dispersal distance observed in males was 467 km [15]. However, physical movement apparently does not necessarily translate into gene flow, as both the estimates of long-term gene flow (effective migrants per generation) among clusters (males: 0.168–1.114; females: 0.107–1.309; Table 3) as well as the inter-regional migration rates, i.e., current gene flow (males: 0.002–0.106; females: 0.002–0.162; Table 4) were low. In the Finnish brown bear population, where the genetic structure decreased relatively rapidly, the estimated number of effective migrants ranged from 1.60–3.63, displaying an increase over time [14]. An even higher number of effective migrants was estimated for the gene flow between Finland and Russian Karelia (7.64, [13]). Contrary to our expectations based on the clear bias in dispersal distance and probability, there was no consistent difference between males and females for the estimated migration rate, and in some instances, female migration rates between three specific regions was even higher than the equivalent male estimates (South Central to South, North to South, and South Central; Table 4).

## Conclusions

In the study of sex-biased dispersal, the use of genetic approaches has been suggested to augment the findings of more ecological methods, e.g. radio-tracking [9, 20, 85]. Our results show that this approach can uncover previously undetected processes, as it allows a larger coverage spatially and greater sample size, especially when using material collected in the course of monitoring schemes. The Scandinavian brown bear population seems to be well on its way towards its prebottleneck state [10], although in Norway population numbers remain low compared to the historical size [10, 86]. Demographic recovery of a population, as observed here, is often associated with increased gene flow [14, 87]. Here, we have shown that the correlation of population and gene flow increase should not be assumed, even in species with long-distance dispersal capabilities, such as the brown bear. Several studies have found that dispersal decisions are condition dependent and consequently dispersal rate and frequency may vary among locations and individuals [88–93]. However, as gene flow is widely accepted to be an important factor in population viability [1] and to ensure the long-term existence of the Scandinavian brown bear population, future studies should try to detangle natural, i.e. species-specific, from anthropogenic causes for the observed patterns, by taking environmental and individual characteristics into account.

## Supporting information

S1 Fig Results of the Bayesian clustering analysis of brown bears in Sweden and Norway with STRUCTURE, processed with Structure Harvester. a) results of the analysis performed on the total dataset; b) results of the analysis of only females; c) results of the analysis of only males.
(PDF)

S2 Fig Barplots of the STRUCTURE analysis of brown bears in Sweden and Norway. Each bear is represented by one bar, the segments of which are sized and colored according to the estimated assignment probability *q* for the given number of clusters, K; the individuals are sorted from south to north. a) results for the analysis of males and females combined (n = 1531) for K = 2 and K = 4; b) results for the analysis of only females (n = 742) for K = 2 and K = 4; c) results for the analysis of only males (n = 789) for K = 3 and K = 4.
(PDF)

S3 Fig Results of the STRUCTURE analysis within clusters of female bears. Results were processed with the help of Cluster Harvester [29]. a) cluster 1, b) cluster 3 and c) cluster 4.
(PDF)

S4 Fig Results of the STRUCTURE analysis within clusters of male bears. Results were processed with the help of Cluster Harvester (Earl & von Holdt 2012). a) cluster three, b) cluster four.
(PDF)

S5 Fig Barplots of the Bayesian clustering analysis of brown bears in Sweden and Norway within previously determined main clusters with STRUCTURE. Each bar equals one bear, the segments of which are sized and colored according to the estimated assignment probability *q* for the given number of subclusters K. Each barplot is ordered according to sampling region, either from south to north (a, b and d) or from west to east (c and e). a) results for the analysis of females in cluster 1 (n = 249) for K = 2 and K = 3; b) results for the analysis of females in cluster 3 (n = 203) for K = 4; c) results for the analysis of females in cluster 4 (n = 48) for K = 3; d) results for the analysis of males in cluster 3 (n = 193) for K = 2 and K = 3; e) results for the analysis of males in cluster 4 (n = 63) for K = 2.
(PDF)

S6 Fig Regional analysis of spatial autocorrelation performed with GenAlEx. Samples were grouped according to sampling location indicated by black circles on the map next to each graph. In the graphs the results for the females is given as a solid black line, the males as a dashed line. The 95% confidence intervals and bootstrap errors are given in the same manner.
(PDF)

S7 Fig Principle coordinate analysis performed for each of the four differentiation estimates, using the combined data. (PDF)

S8 Fig Number of migrant brown bears per generation among the four genetic clusters in Scandinavia, estimated by the private allele method and for increasing sample size. Given are the estimates for females (▲), males (■) and both sexes combined (●).
(PDF)

S9 Fig Probability for the run used in the final results for the estimation of self-recruitment and migration among the different regions in Scandinavia, estimated using BAYESASS 3.0 and the script published by Meirmans [57]. a) shows the result for the male bears, b) shows the result for the female bears.
(PDF)

S10 Fig Consistency in the estimates of the nonmigrant proportion in ten independent runs of BAYESASS for male Scandinavian brown bears. (PDF)

S11 Fig Consistency in the estimates of the nonmigrant proportion in ten independent runs of BAYESASS for female Scandinavian brown bears. (PDF)
