# Supplementary material for: Sex-specific genetic analysis indicates low correlation between demographic and genetic connectivity in the Scandinavian brown bear (Ursus arctos)
Source: PLoS One. 2017 Jul 3;12(7):e0180701. doi: 10.1371/journal.pone.0180701 (PMC5495496; doi:10.1371/journal.pone.0180701)
Supplement: S1 Fig — a) results of the analysis performed on the total dataset; b) results of the analysis of only females; c) results of the analysis of only males. (PDF) [file pone.0180701.s001.pdf]

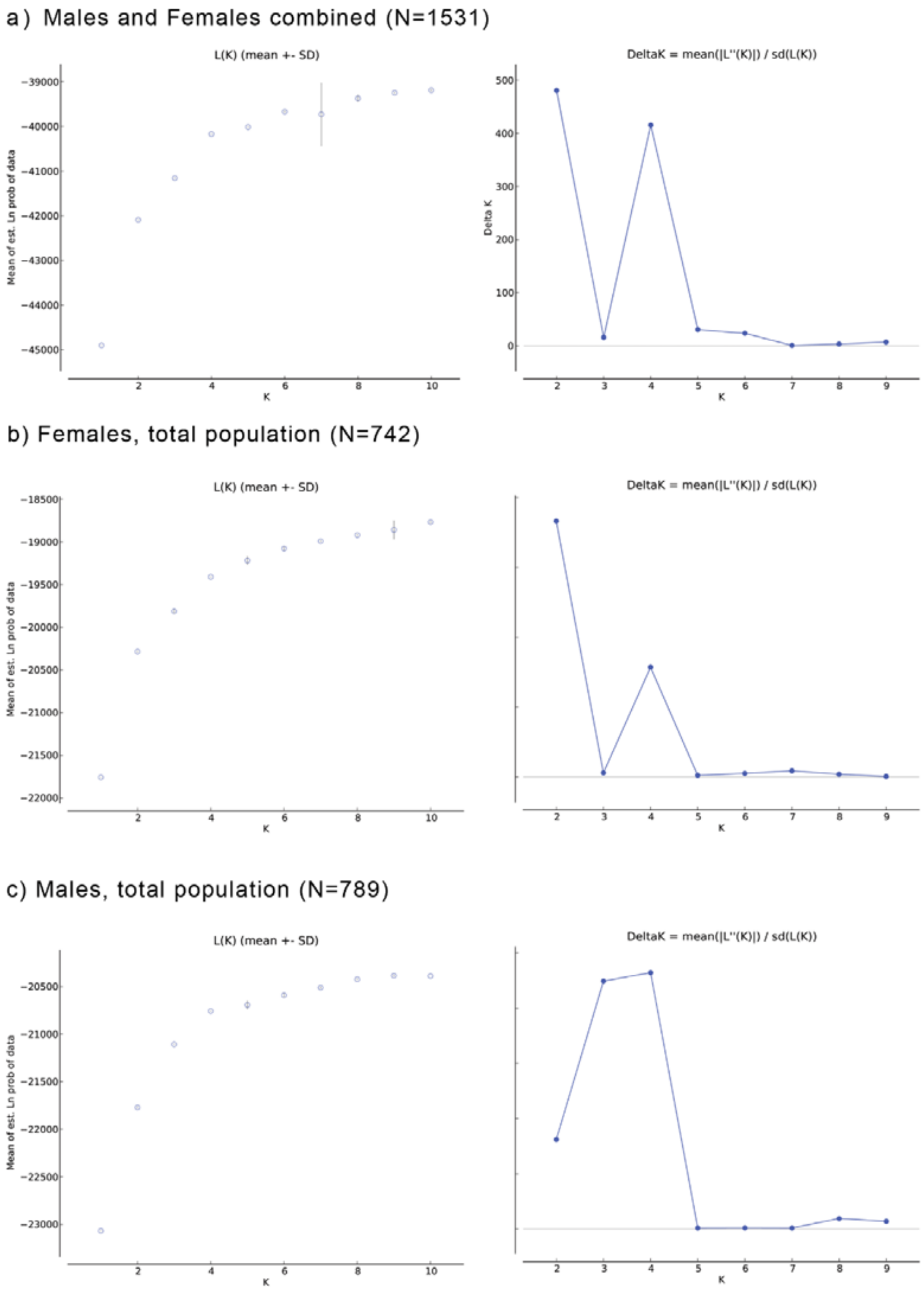

S1 Fig. Results of the Bayesian clustering analysis of brown bears in Sweden and Norway with STRUCTURE, processed with Structure Harvester. a) results of the analysis performed on the total dataset; b) results of the analysis of only females; c) results of the analysis of only males.
