# Supplementary material for: Sex-specific genetic analysis indicates low correlation between demographic and genetic connectivity in the Scandinavian brown bear (Ursus arctos)
Source: PLoS One. 2017 Jul 3;12(7):e0180701. doi: 10.1371/journal.pone.0180701 (PMC5495496; doi:10.1371/journal.pone.0180701)
Supplement: S2 Fig — Each bear is represented by one bar, the segments of which are sized and colored according to the estimated assignment probability q for the given number of clusters, K; the individuals are sorted from south to north. a) results for the analysis of males and females combined (n = 1531) for K = 2 and K = 4; b) results for the analysis of only females (n = 742) for K = 2 and K = 4; c) results for the analysis of only males (n = 789) for K = 3 and K = 4. (PDF) [file pone.0180701.s002.pdf]

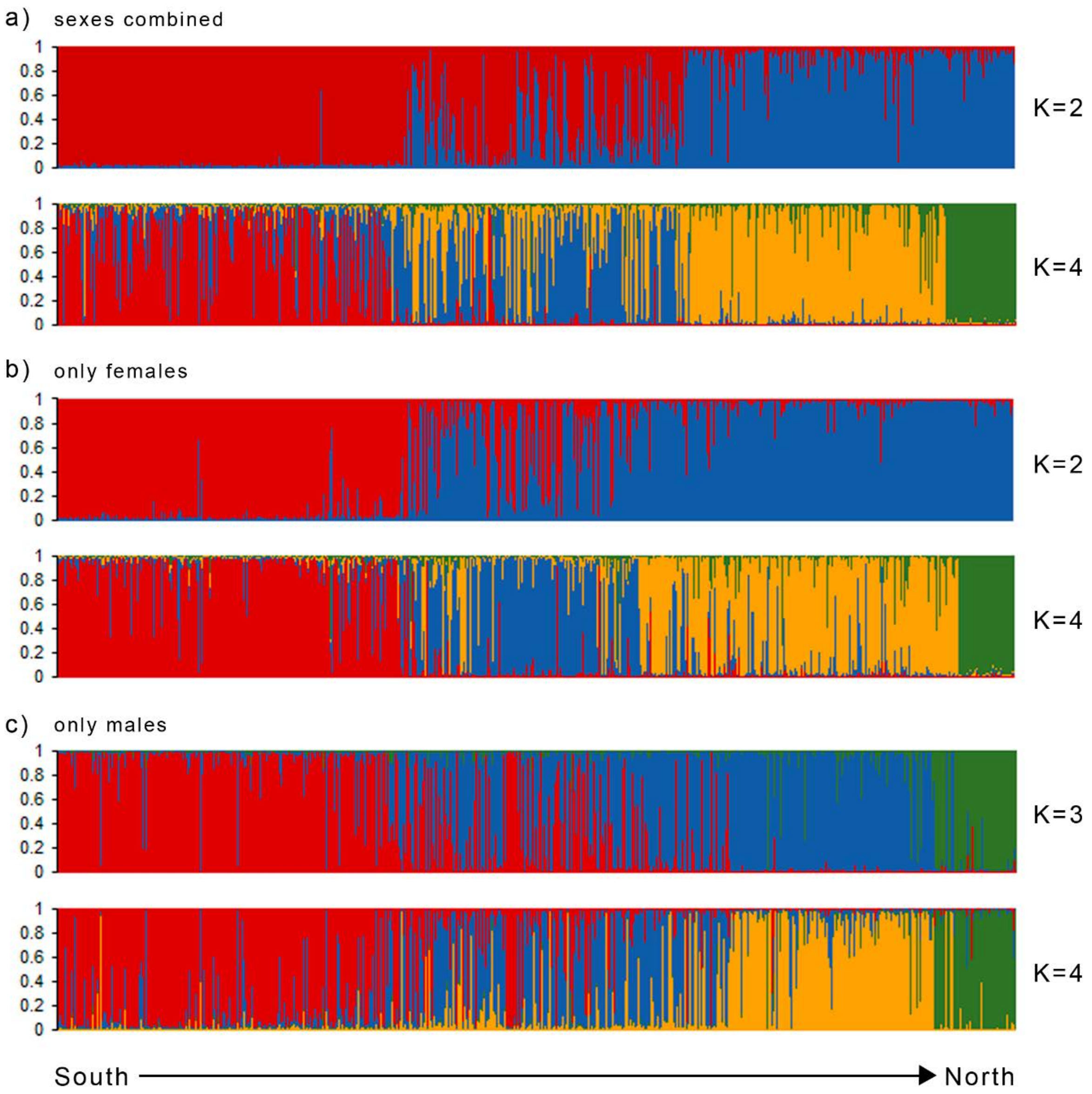

S2 Fig. Barplots of the STRUCTURE analysis of brown bears in Sweden and Norway. Each bear is represented by one bar, the segments of which are sized and colored according to the estimated assignment probability *q* for the given number of clusters, K; the individuals are sorted from south to north. a) results for the analysis of males and females combined (n=1531) for K=2 and K=4; b) results for the analysis of only females (n=742) for K=2 and K=4; c) results for the analysis of only males (n=789) for K=3 and K=4.
