# Supplementary material for: Sex-specific genetic analysis indicates low correlation between demographic and genetic connectivity in the Scandinavian brown bear (Ursus arctos)
Source: PLoS One. 2017 Jul 3;12(7):e0180701. doi: 10.1371/journal.pone.0180701 (PMC5495496; doi:10.1371/journal.pone.0180701)
Supplement: S3 Fig — Results were processed with the help of Cluster Harvester [29]. a) cluster 1, b) cluster 3 and c) cluster 4. (PDF) [file pone.0180701.s003.pdf]

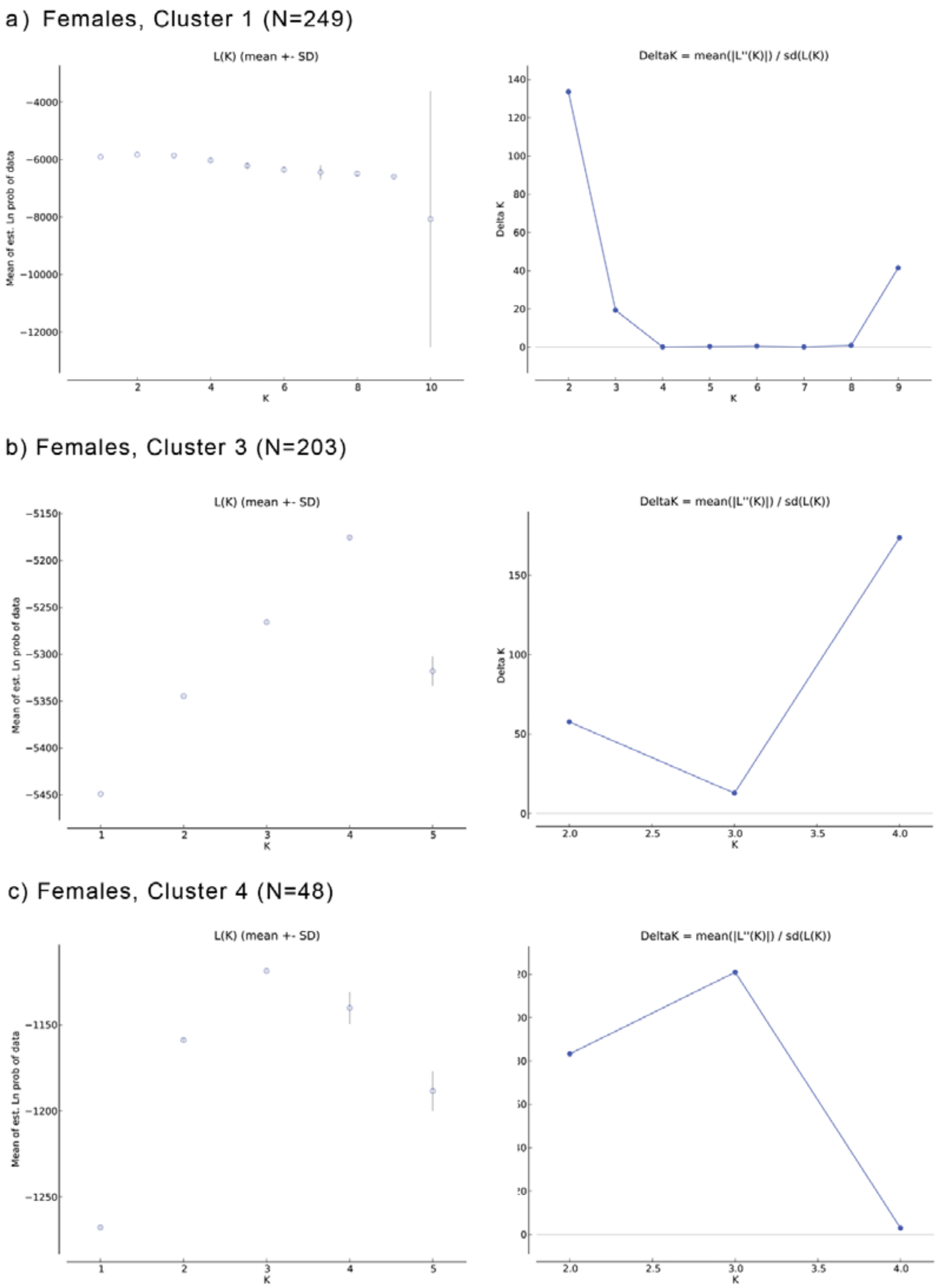

S3 Fig. Results of the STRUCTURE analysis within clusters of female bears. Results were processed with the help of Cluster Harvester [29]. a) cluster 1, b) cluster 3 and c) cluster 4.
