# Supplementary material for: Sex-specific genetic analysis indicates low correlation between demographic and genetic connectivity in the Scandinavian brown bear (Ursus arctos)
Source: PLoS One. 2017 Jul 3;12(7):e0180701. doi: 10.1371/journal.pone.0180701 (PMC5495496; doi:10.1371/journal.pone.0180701)
Supplement: S4 Fig — Results were processed with the help of Cluster Harvester (Earl & von Holdt 2012). a) cluster three, b) cluster four. (PDF) [file pone.0180701.s004.pdf]

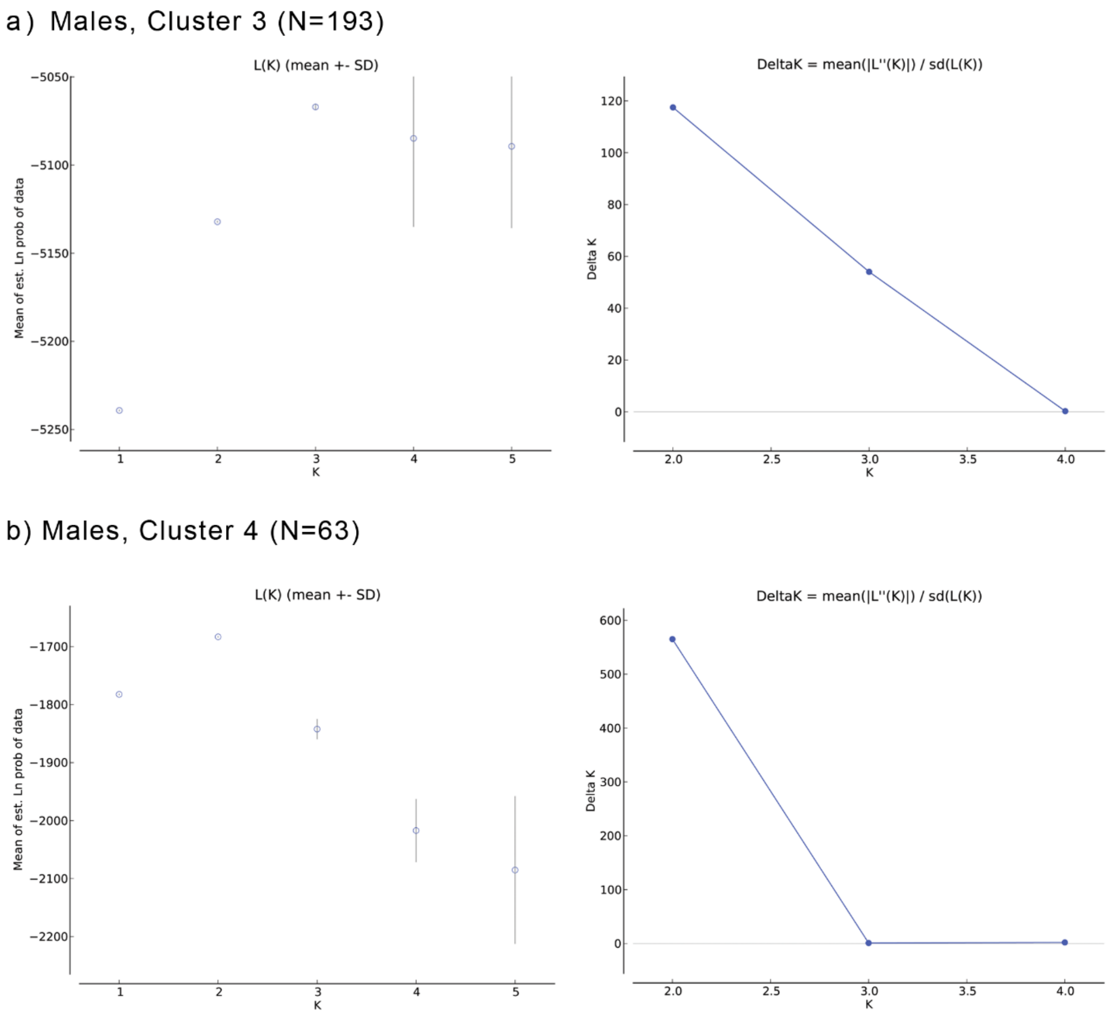

S4 Fig. **Results of the STRUCTURE analysis within clusters of male bears.** Results were processed with the help of Cluster Harvester (Earl & von Holdt 2012). a) cluster three, b) cluster four.
