# Supplementary material for: Sex-specific genetic analysis indicates low correlation between demographic and genetic connectivity in the Scandinavian brown bear (Ursus arctos)
Source: PLoS One. 2017 Jul 3;12(7):e0180701. doi: 10.1371/journal.pone.0180701 (PMC5495496; doi:10.1371/journal.pone.0180701)
Supplement: S5 Fig — Each bar equals one bear, the segments of which are sized and colored according to the estimated assignment probability q for the given number of subclusters K. Each barplot is ordered according to sampling region, either from south to north (a, b and d) or from west to east (c and e). a) results for the analysis of females in cluster 1 (n = 249) for K = 2 and K = 3; b) results for the analysis of females in cluster 3 (n = 203) for K = 4; c) results for the analysis of females in cluster 4 (n = 48) for K = 3; d) results for the analysis of males in cluster 3 (n = 193) for K = 2 and K = 3; e) results for the analysis of males in cluster 4 (n = 63) for K = 2. (PDF) [file pone.0180701.s005.pdf]

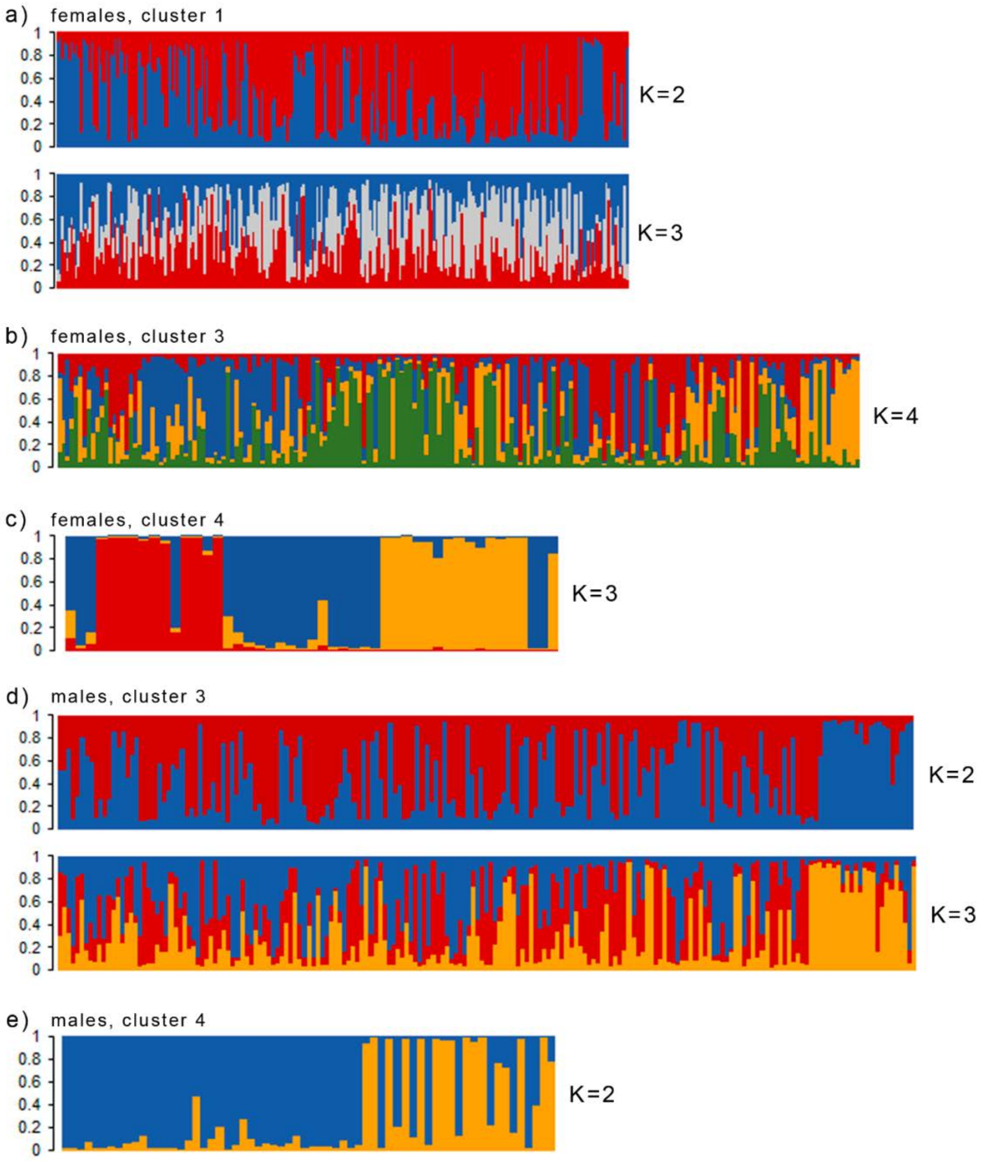

S5 Fig. Barplots of the Bayesian clustering analysis of brown bears in Sweden and Norway within previously determined main clusters with STRUCTURE. Each bar equals one bear, the segments of which are sized and colored according to the estimated assignment probability *q* for the given number of subclusters K. Each barplot is ordered according to sampling region, either from south to north (a, b and d) or from west to east (c and e). a) results for the analysis of females in cluster 1 (n=249) for K=2 and K=3; b) results for the analysis of females in cluster 3 (n=203) for K=4; c) results for the analysis of females in cluster 4 (n=48) for K=3; d) results for the analysis of males in cluster 3 (n=193) for K=2 and K=3; e) results for the analysis of males in cluster 4 (n=63) for K=2.
