# Supplementary material for: Sex-specific genetic analysis indicates low correlation between demographic and genetic connectivity in the Scandinavian brown bear (Ursus arctos)
Source: PLoS One. 2017 Jul 3;12(7):e0180701. doi: 10.1371/journal.pone.0180701 (PMC5495496; doi:10.1371/journal.pone.0180701)
Supplement: S6 Fig — Samples were grouped according to sampling location indicated by black circles on the map next to each graph. In the graphs the results for the females is given as a solid black line, the males as a dashed line. The 95% confidence intervals and bootstrap errors are given in the same manner. (PDF) [file pone.0180701.s006.pdf]

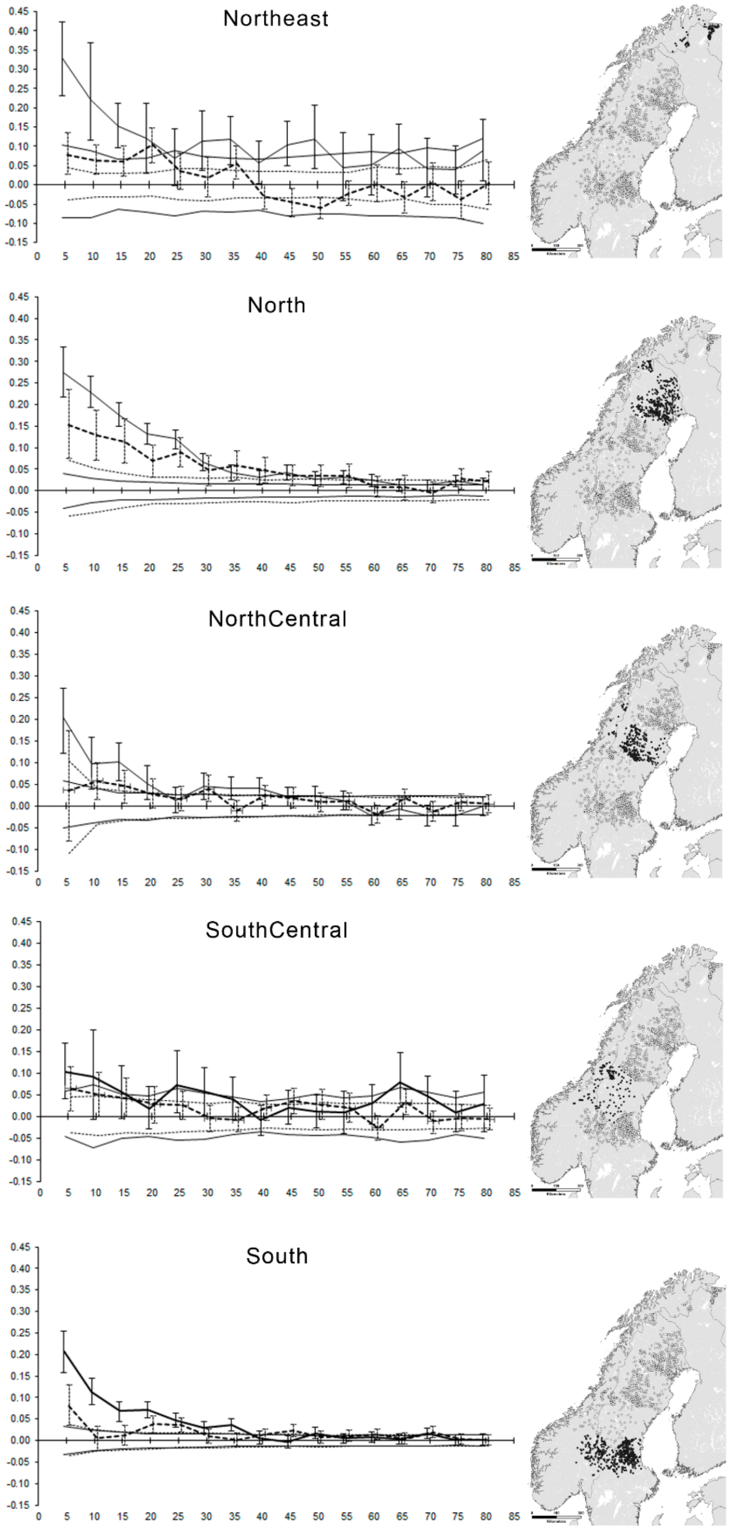

S6 Fig. Regional analysis of spatial autocorrelation performed with GenAlEx. Samples were grouped according to sampling location indicated by black circles on the map next to each graph. In the graphs the results for the females is given as a solid black line, the males as a dashed line. The 95 % confidence intervals and bootstrap errors are given in the same manner.
