# Supplementary material for: Sex-specific genetic analysis indicates low correlation between demographic and genetic connectivity in the Scandinavian brown bear (Ursus arctos)
Source: PLoS One. 2017 Jul 3;12(7):e0180701. doi: 10.1371/journal.pone.0180701 (PMC5495496; doi:10.1371/journal.pone.0180701)
Supplement: S7 Fig — (PDF) [file pone.0180701.s007.pdf]

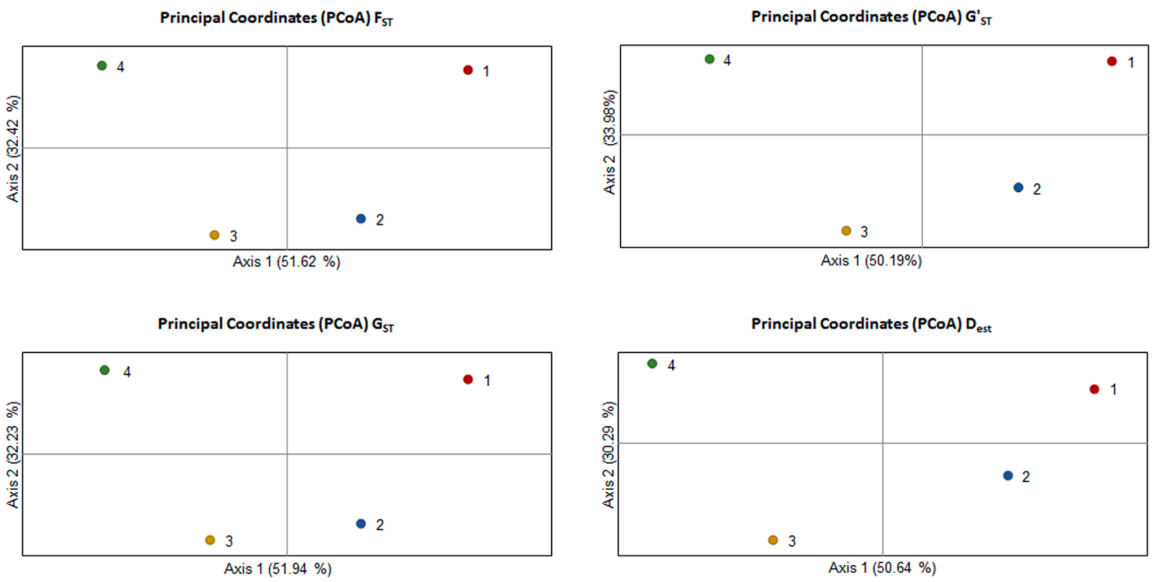

S7 Fig. Principle Coordinate Analysis performed for each of the four differentiation estimates, using the combined data.
