# Supplementary material for: Sex-specific genetic analysis indicates low correlation between demographic and genetic connectivity in the Scandinavian brown bear (Ursus arctos)
Source: PLoS One. 2017 Jul 3;12(7):e0180701. doi: 10.1371/journal.pone.0180701 (PMC5495496; doi:10.1371/journal.pone.0180701)
Supplement: S8 Fig — Given are the estimates for females (▲), males (■) and both sexes combined (●). (PDF) [file pone.0180701.s008.pdf]

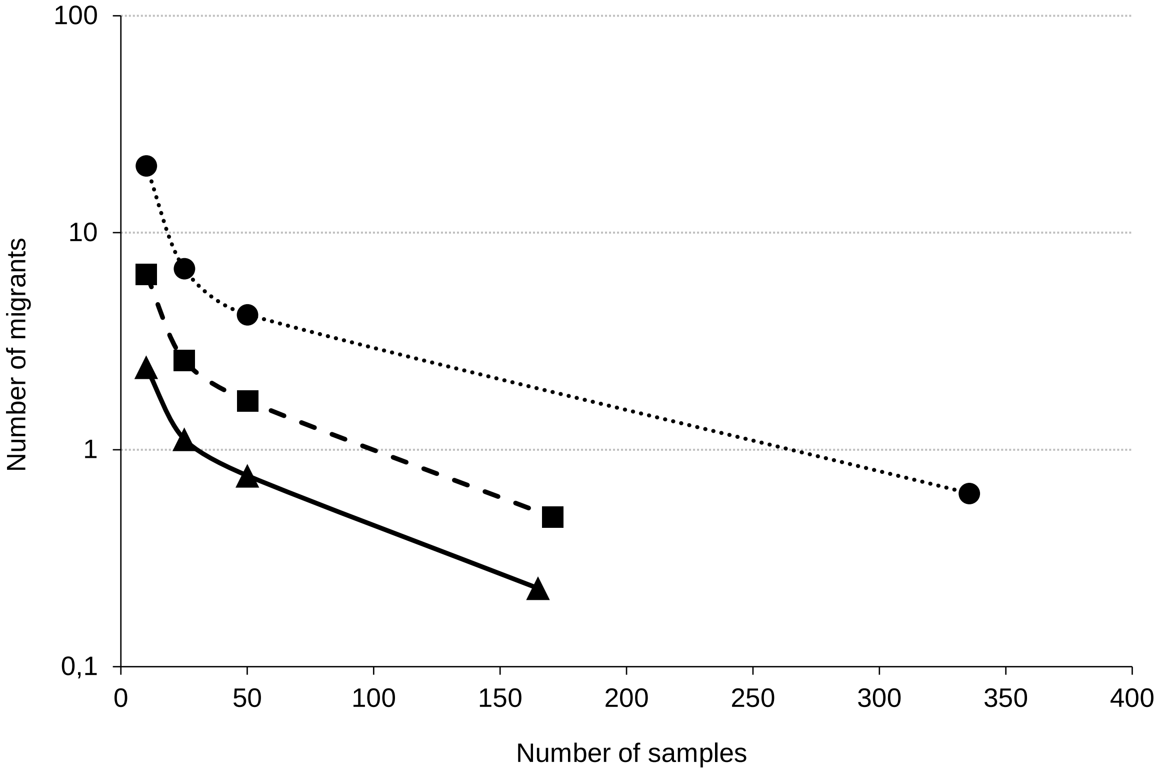

S8 Fig. Number of migrant brown bears per generation among the four genetic clusters in Scandinavia, estimated by the private allele method and for increasing sample size. Given are the estimates for females (▲), males (■) and both sexes combined (●).
