# Supplementary material for: Sex-specific genetic analysis indicates low correlation between demographic and genetic connectivity in the Scandinavian brown bear (Ursus arctos)
Source: PLoS One. 2017 Jul 3;12(7):e0180701. doi: 10.1371/journal.pone.0180701 (PMC5495496; doi:10.1371/journal.pone.0180701)
Supplement: S9 Fig — a) shows the result for the male bears, b) shows the result for the female bears. (PDF) [file pone.0180701.s009.pdf]

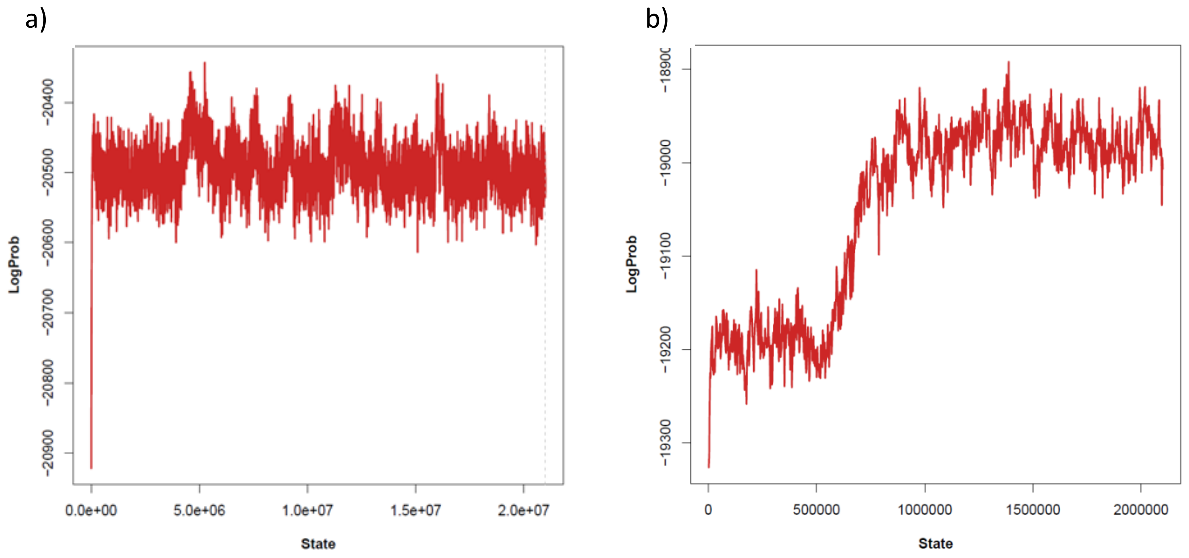

S9 Fig. Probability for the run used in the final results for the estimation of self-recruitment and migration among the different regions in Scandinavia, estimated using BAYESASS 3.0 and the script published by Meirmans [57]. a) shows the result for the male bears, b) shows the result for the female bears.
